# Arsenic Disruption of DNA Damage Responses—Potential Role in Carcinogenesis and Chemotherapy

**DOI:** 10.3390/biom5042184

**Published:** 2015-09-24

**Authors:** Clarisse S. Muenyi, Mats Ljungman, J. Christopher States

**Affiliations:** 1 Department of Pharmacology and Toxicology, University of Louisville School of Medicine, Louisville, KY 40292, USA; E-Mail: clarisse.muenyi@louisville.edu; 2 Departments of Radiation Oncology and Environmental Health Sciences, University of Michigan, Ann Arbor, MI 48109-2800, USA; E-Mail: ljungman@med.umich.edu; 3 Department of Pharmacology and Toxicology, University of Louisville School of Medicine, Louisville, KY 40292, USA

**Keywords:** arsenic, DNA repair, DNA damage response, ubiquitination, XPC, MSH2

## Abstract

Arsenic is a Class I human carcinogen and is widespread in the environment. Chronic arsenic exposure causes cancer in skin, lung and bladder, as well as in other organs. Paradoxically, arsenic also is a potent chemotherapeutic against acute promyelocytic leukemia and can potentiate the cytotoxic effects of DNA damaging chemotherapeutics, such as cisplatin, *in vitro*. Arsenic has long been implicated in DNA repair inhibition, cell cycle disruption, and ubiquitination dysregulation, all negatively impacting the DNA damage response and potentially contributing to both the carcinogenic and chemotherapeutic potential of arsenic. Recent studies have provided mechanistic insights into how arsenic interferes with these processes including disruption of zinc fingers and suppression of gene expression. This review discusses these effects of arsenic with a view toward understanding the impact on the DNA damage response.

Arsenic, a well-known environmental hazard, has a long history as a carcinogen and chemotherapeutic [1,2]. Arsenic in drinking water is the most common source of arsenic exposure [3]. The diet is also a major source of As, particularly at levels below 10 µg As/L in water [4]. Several studies have demonstrated that chronic exposures to low doses (<100 µg/L) of arsenic in drinking water is associated with increased risk of skin, lung, bladder and kidney cancers [5,6] in humans; liver cancer is associated with higher exposure (>250 µg/L) [7]. There is a large body of literature supporting a variety of mechanisms for arsenic carcinogenesis including DNA repair inhibition, gene expression alterations via epigenetic modifications, stem cell population expansion [8] and aneuploidogenesis [9,10]. Arsenic also is a proven therapeutic for treating acute promyelocytic leukemia, and has potential benefits for treating other leukemias, as well as solid tumors. This review is focused on exploring the role that arsenic disruption of the DNA damage response may play in both carcinogenesis and chemotherapy.

Chronic arsenic exposure including an *in utero* period will induce a variety of internal tumors in mice [11]. However, exposure to arsenic alone is not sufficient to induce cancer in adult animals but rather, acts as a co-carcinogen to enhance tumorigenesis, for example UV induced squamous cell carcinoma in hairless mice [12,13,14,15]. This effect of arsenic is similar to tumor promoters such as phorbol esters [16]. Furthermore, arsenic enhances phorbol ester promoted skin carcinogenesis in Tg.AC mice (harbor v-Ha-ras) although arsenic has no effect when given alone to these animals [17]. Thus, arsenic alone is not sufficient to induce skin cancer in mice but in the presence of other genotoxic agents, such as UV light, arsenic causes increased tumorigenesis.

Interference with DNA repair activities has long been implicated as a mechanism of arsenic’s carcinogenic effect in humans when exposed through contaminated drinking water [18,19]. Arsenic interference with DNA repair is expected to promote mutation of key tumor suppressor genes such as tumor protein P53 (TP53) in patients exposed to arsenic, leading to increased risk of bladder cancer [20,21]. However, bladder cancers from patients with high arsenic exposure exhibited a low rate of TP53 mutations present in patients who had used hair dyes [20] suggesting that arsenic exposure did not promote mutagenesis in humans. Similarly, TP53 mutagenesis is known to be a driver of sunlight induced squamous cell carcinoma [22] and the rodent studies discussed above would suggest an arsenic-induced increase in UV-induced mutagenesis. However, studies of TP53 mutations in skin tumors associated with arsenic exposure in humans showed either no mutations [23,24] or mutations not associated with UV light exposure [25]. Chronic exposure to low arsenite (0.1 µM) led to poly (ADP-ribosyl)ation of TP53 and poly (ADP-ribose) polymerase 1 (PARP1) in HaCaT cells [26]. It is well established that, following DNA damage by a genotoxic agent, activated TP53 transcriptionally induces expression of p21 (cyclin dependent kinase inhibitor 1A, CDKN1A) which in turn causes cell cycle arrest allowing for damaged DNA to be repaired before continuing the cell cycle. However, decreased expression of p21 mRNA and protein were observed following concomitant arsenic exposure, suggesting the inactivation of TP53 function following its poly (ADP-ribosyl)ation by arsenite [26]. As discussed below, later reports showed that arsenite inhibited PARP1 activity in HaCaT cells [27,28]. Other mechanisms of abrogating TP53 function must be operative if indeed PARP1 is inhibited by arsenite. Indeed, earlier reports in a variety of cell types showed that arsenite induced TP53 and CDKN1A [29,30,31]. In addition to its effect on TP53 and the cell cycle, recent evidence points to inhibition of base excision repair (BER) and nucleotide excision repair (NER).

Exposure to arsenic and other oxidative stress inducers can result in oxidative DNA damage [32,33,34,35,36,37] that is typically repaired by the BER pathway. In an effort to elucidate the mechanism of arsenic-induced carcinogenesis, Ebert *et al.* [38] studied the effect of arsenic and its metabolites on BER pathway in human A549 lung cancer cells. Arsenite at 10 µM or greater significantly decreased the levels of DNA ligase IIIa (LIG3), dimethylarsinic acid (DMA^V^) starting at 5 µM significantly decreased human 8-oxoguanine DNA glycosylase-1 (OGG1) activity. However, higher concentrations were required for arsenite and monomethylarsonic acid (MMA^V^) to induce similar effects on OGG1. MMA^V^ and arsenite at low concentrations (2.5 and 5 µM, respectively) significantly decreased X-ray cross complementing protein 1 (XRCC1) levels [38]. These data indicate that arsenic or its metabolites impair BER pathway. Osmond *et al.* [39] also demonstrated that neonatal mice exposed to arsenic contaminated lactation milk showed significant dose- and age-dependent decrease in apurinic/apyrimidinic (abasic) endonuclease (APEX1), DNA ligase 1 (LIG1), LIG3, OGG1, PARP1, and DNA polymerase β (POLB) mRNAs, further supporting impairment of the BER pathway by arsenic.

Poly (ADP-ribosyl)ation of nuclear proteins following DNA damage is catalyzed by PARP1. Poly (ADP-ribosyl)ation is required for the dissociation of nuclear proteins so that base excision repair proteins can gain access to the damaged site and repair the DNA. Hartwig *et al.* demonstrated that very low concentration of arsenite (10 nM) inhibited PARP1 in HeLa cells and significantly increased the number of DNA strand breaks by H_2_O_2_ [40]. The molecular target of arsenic is the zinc finger motif in DNA repair proteins, such as xeroderma pigmentosum group A (XPA) [41] and PARP1 [42]. As a consequence, repair of oxidative DNA damage is inhibited [27]. Inhibition of zinc finger dependent PARP1 activity by arsenite was observed in HaCaT cells treated with UV, and this effect was diminished by adding zinc (II) ions [27,28]. These studies support the idea that arsenic interaction with zinc finger proteins involved in DNA repair causes impaired DNA repair and contributes to carcinogenesis.

Arsenic is a chemotherapeutic commonly used against hematological and solid tumors. Arsenic trioxide was approved by the Food and Drug Administration in 2001 for the treatment of all-*trans* retinoic acid (ATRA) resistant acute promyelocytic leukemia [43]. Arsenic trioxide induces apoptosis in various forms of solid cancer cells *in vitro* [44,45,46], and it inhibits the growth of orthotopic metastatic prostate cancer and peritoneal metastatic ovarian cancer [42,47]. Arsenic is more effective as a chemotherapeutic against solid tumors when used in combination with other agents such as hyperthermia, radiation, cisplatin, adriamycin, doxorubicin, and etoposide [45,48,49]. Results of experiments with metastatic cisplatin-resistant human epithelial ovarian cancer xenografts established in nude mice and subjected to intraperitoneal chemotherapeutic treatment combining cisplatin (3 mg/kg) ± sodium arsenite (26 mg/kg) ± hyperthermia (37 or 43 °C) for 1 h revealed that cisplatin alone (±hyperthermia) induced TP53, XPC and XPA and suppressed MSH2 [50]. The data indicate that the induced DNA damage response of these cisplatin-resistant cells was part of the resistance mechanism. XPC is a DNA damage recognition protein in the global genomic nucleotide excision repair pathway (GG-NER) [51] and MSH2 is a key player in DNA mismatch repair (MMR). MMR deficiency is associated with increased risk for colorectal cancer [52] and ovarian cancer [53]. However, once a cancer has formed, MMR loss is correlated with cisplatin resistance because MMR proteins can bind to lesions and block access by DNA damage recognition proteins of other DNA repair mechanisms and MMR can enter a futile repair cycle inducing apoptosis [54]. Sodium arsenite co-treatment prevented XPC induction by cisplatin, thus, inhibiting the GG-NER pathway. Additionally, arsenite maintained higher levels of MSH2, thus maintaining functional MMR and sensitivity to cisplatin in the tumors. Consistent with the idea that repair of platinum adducts is impaired, arsenite also enhanced the accumulation of platinum in the tumors [50]. TP53 transcriptionally regulates XPC following DNA damage [55,56,57,58]. Nollen *et al.* [59] demonstrated that arsenic inhibits NER by suppressing XPC in fibroblasts. We investigated the role of TP53 in epithelial ovarian cancer response to cisplatin, sodium arsenite (20 µM) and hyperthermia (39 °C) in wild-type TP53 expressing (A2780, A2780/CP70, OVCA 420, OVCA 429, and OVCA 433), TP53-null (SKOV-3) and TP53-mutant (OVCA 432 and OVCAR-3) ovarian cancer cells [60]. Arsenite alone or in combination with hyperthermia selectively sensitized TP53-expressing cells to cisplatin [60]; arsenite sensitization effect was abrogated by TP53 siRNA. Furthermore, we demonstrated that arsenite ± hyperthermia decreased XPC in TP53-expressing ovarian cancer cells. XPC siRNA increased cisplatin sensitivity in TP53-expressing cells. Additionally, we showed that hyperthermia ± arsenite increased both cellular and DNA platinum levels in TP53-expressing cells. These data indicate that arsenite sensitizes TP53-expressing ovarian cancer cells to cisplatin by increasing cellular and DNA platinum accumulation and by attenuation of XPC, a GG-NER protein [60]. We further demonstrated that co-treatment of TP53-expressing ovarian cancer cells with cisplatin + arsenite (20 µM) at 39 °C induced a pseudo-G1 associated apoptotic cell death [61]. Cells at 36 h post treatment appeared to accumulate in G2/M compartment by flow cytometry; decreased protein level of cyclin A and stabilization of cyclin B suggested mitotic arrest. However, mitotic index was very low and histone H3 Ser10 phosphorylation was undetectable, indicating that cells were not accumulating in mitosis. Failure of BUB1 mitotic checkpoint serine/threonine kinase B (BUB1B aka BUBR1) phosphorylation following cisplatin + arsenite (20 µM) at 39 °C treatment suggested disruption of mitotic checkpoint. G1 cell cycle arrest at 36 h post treatment with cisplatin, sodium arsenite and hyperthermia was confirmed by increased accumulation of P21 (CDKN1A), decreased retinoblastoma 1 (RB1) phosphorylation and stabilization of cyclin E. These data indicate that arsenite and hyperthermia disrupted the mitotic checkpoint in TP53-expressing ovarian cancer cells; undivided cells exited mitosis and accumulated in pseudo-G1 with 2C DNA content and subsequently underwent apoptosis. Thus, arsenite can interfere with the DNA damage response not only by disrupting induction of DNA repair gene expression but also by disruption of cell cycle controls.

In unpublished preliminary studies to understand more completely the impact of arsenite on DNA damage response, we used Bru-seq to identify transcriptional effects of an acute arsenite exposure. Bru-seq is a new technique that allows one to quantify genome wide nascent transcription [62]. We treated A2780/CP70 ovarian cancer cells with 5 µM arsenite for 1 h followed by a washout and performed Bru-seq analysis of nascent transcripts 5 h after treatment. Arsenite exposed cells showed significant inhibition of a multitude of transcripts; DNA repair pathways and ubiquitin mediated proteolysis pathways were among the most affected.

Among nucleotide excision repair genes with decreased transcription were two genes encoding proteins involved in DNA damage recognition. *RAD23 homolog B* (*RAD23B*) which forms a heterodimer with XPC was down two-fold, and *damage-specific DNA binding protein 2* (*DDB2*) which also plays a role in damage recognition and interacts with XPC/RAD23B was down six-fold. Transcription of several genes encoding components of the transcription factor IIH (TFIIH) complex which plays a role in both nucleotide excision repair and transcription initiation also was decreased; *excision repair cross-complementation group 3* (*ERCC3*) and general *transcription factor IIH*, *polypeptide 5* (*GTF2H5*) were down four-fold and three-fold respectively, suggesting decreased TFIIH. These results are consistent with a decreased ability to repair cisplatin adducts and UV photoproducts and thus, with increased sensitivity to cisplatin therapy and to UV-induced mutagenesis.

Ubiquitin mediated proteolysis is critical to cell cycle regulation, and disruption of gene expression required for this regulation will abolish the cell cycle regulatory response to DNA damage. Transcription of *anaphase promoting complex subunits 7* (*ANAPC7*) and *10* (*ANAPC10*), which are core subunits of the anaphase promoting complex/cyclosome (APC/C) were down seven-fold and five-fold, respectively. The anaphase promoting complex/cyclosome (APC/C) regulates progression through mitosis and entry into G1 phase. Disruption of the APC/C by arsenite is consistent with our observations of disruption of mitotic progression by arsenite [29,30,31,61]. Many of the DNA repair genes also encode proteins with ubiquitin ligase activity, such as *DDB2* and *Fanconi Anemia, complementation group L* (*FANCL*, down seven-fold). FANCL is an ubiquitin ligase protein that mediates monoubiquitination of FANCD2 and FANCI, key steps in the interstrand crosslink repair pathway. Suppression of interstrand crosslink repair also would sensitize cells to cisplatin, consistent with our observations discussed above [50,60]. DDB2 interacts with the DCX (DDB1-CUL4-X-box) E3 ubiquitin ligase to ubiquitinylate histones H3 and H4, as well as XPC, at sites of DNA damage during NER. Thus, decreased expression of these genes would be expected to interfere with the DNA repair responses to both interstrand crosslinks and bulky adducts.

The broad suppression of transcription observed in the Bru-seq experiment suggests the possibility that major transformation of chromatin structure through an epigenetic effect may have occurred. That arsenic causes epigenetic changes is well established [63]. Global changes in post-translational modification of histones occurred in A549 human lung cells exposed to arsenite for 24 h or 7 days [64]. The changes included an increase in H3K4me3 suggesting that global repression of transcription may be occurring. Thus, epigenetic changes induced by arsenic exposure may underlie the changes observed in the various DNA repair gene systems.

Our studies of co-treatment of ovarian cancer cells with arsenite and cisplatin indicated that TP53 signaling was disrupted. The Bru-seq data indicate that transcription of *MDM2*, encoding an E3 ubiquitin protein ligase that regulates TP53 levels, was suppressed 10-fold in arsenite exposed cells. This decrease in *MDM2* transcription is consistent with the observed increase of TP53. The transcription of signaling kinase genes upstream of TP53 also was decreased: *ATM serine/threonine kinase* (*ATM*) was down two-fold; *ATR serine/threonine kinase* (*ATR*), three-fold; and *checkpoint kinase 1* (*CHEK1*), three-fold. Thus, arsenite exposure suppresses transcription of the major DNA damage signaling genes likely contributing to disrupted DNA damage responses.

In summary, arsenic interferes with the DNA damage response at multiple levels, negatively impacting DNA repair capability and cell cycle control mechanisms as illustrated in Figure 1. Arsenite directly disrupts function of zinc finger proteins, such as XPA and PARP1 decreasing DNA repair capacity. Arsenite also suppresses expression of key genes in DNA repair and ubiquitin mediated proteolysis pathways. Suppression of DNA repair gene expression directly attenuates the DNA repair response to DNA damage. Suppression of the ubiquitin pathways attenuates both DNA repair and cell cycle regulatory processes. These effects of arsenic exposure likely contribute to its roles as both a carcinogen and a potential chemotherapeutic.

**Figure 1:**
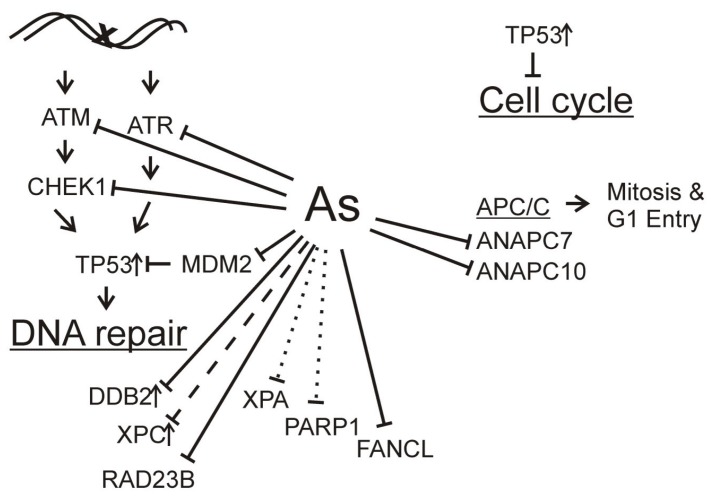
Arsenic inhibition of DNA damage response. DNA damage normally leads to activation of signaling kinases converging through TP53 stimulating DNA repair gene expression and inhibition of cell cycle. Arsenite inhibits transcription (solid lines) of signaling kinase genes (ATM, ATR, CHEK1), MDM2, and downstream DNA repair genes DDB2 and RAD23B. Expression of XPC also is inhibited by arsenite (dashed line), as is function of XPA and PARP1 by displacing zinc from the zinc fingers in these proteins (dotted lines). These effects likely contribute to decreased nucleotide excision repair. FANCL transcription inhibition may lead to decreased interstrand crosslink repair. Arsenite also inhibits transcription of ANAPC7 and ANAPC10, components of the anaphase promoting complex/cyclosome (APC/C) consistent with arsenite disruption of mitotic progression.
